# Analysis of microRNA expression reveals convergent evolution of the molecular control of diapause in annual killifishes

**DOI:** 10.3389/fgene.2025.1583989

**Published:** 2025-07-03

**Authors:** Emanuel Barth, Mario Baumgart, Luca Dolfi, Rongfeng Cui, Marco Groth, Roberto Ripa, Aurora Savino, Dario Riccardo Valenzano, Matthias Platzer, Manja Marz, Alessandro Cellerino

**Affiliations:** ^1^ Bioinformatics/High Throughput Analysis, Friedrich Schiller University Jena, Jena, Germany; ^2^ Bioinformatics Core Facility Jena, Friedrich Schiller University Jena, Jena, Germany; ^3^ Leibniz Institute on Aging - Fritz Lipmann Institute (FLI), Jena, Germany; ^4^ Laboratory of Biology, Scuola Normale Superiore, Pisa, Italy; ^5^ School of Ecology, Sun Yat-sen University, Guangzhou, China; ^6^ Human Technopole, Milano, Italy; ^7^ Max Planck Institute for Biology of Ageing, Cologne, Germany

**Keywords:** *N. furzeri*, killifish, development, MicroRNAs, diapause

## Abstract

**Background:**

Diapause is a condition of developmental arrest in anticipation of adverse environmental conditions present in many diverse taxa. Diapause is a key adaptation that enabled the colonization of ephemeral habitats subject to the alternation of dry and wet seasons by annual killifishes. Upon desiccation of the ponds, killifish embryos remain vital but quiescent in the clay, where they can survive months or even years. Diapause can occur at three different developmental stages, but Diapause II (DII), which occurs during somitogenesis, is the primary point of developmental arrest. Physiologically, Diapause II is associated with the arrest of the cell cycle in G1 and deeply reduced oxygen consumption and protein synthesis. However, diapause is not obligatory, and some embryos can go through an alternative developmental pathway into direct development, skipping one or more diapauses. The precise molecular mechanisms that regulate entry and exit from diapause are beginning to be investigated, but this knowledge is yet fragmentary. Diapause has evolved independently several times in killifish clades from Africa and South America, enabling identifying possible molecular determinants of diapause by comparative expression analysis. MicroRNAs are small RNAs that represent central nodes in the control of gene expression at the post-transcriptional level and are involved in many developmental processes. Here, we compare microRNA expression profiles of annual killifishes during DII with non-annual killifish in a comparable stage of morphological development.

**Results:**

We used smallRNA-Seq to quantify microRNA expression from four annual- and four non-annual killifish species from three independent clades and from direct-developing embryos of the annual killifish *Nothobranchius furzeri*. We analyzed the expression of broadly conserved microRNAs and microRNAs that appear to have evolved in the killifish lineage. We found several microRNAs that showed convergent regulation in the three different clades, and for some microRNAs also a phenomenon of switch in the prevalent form between 3p and 5p or *vice versa* was noted. In addition, we detected a significant overlap between the microRNA regulation during diapause and aging. Particularly interesting is the regulation of the miR-430 family. These microRNAs represent the second most expressed microRNA family in the killifish embryos, and diapause is associated with dramatic downregulation of the prevalent 3p form and upregulation of the minor 5p form. Members of the miR-430 family are contained in a large repetitive cluster whose organization is variable among teleosts. Analysis of recently sequenced 45 low-coverage killifish genomes revealed that the miR-430 locus contains a lower number of copies in annual-as opposed to non-annual killifish.

**Conclusion:**

The Evolution of diapause is reflected in the convergent evolution of microRNA regulation in killifishes. A prominent feature is a dramatic downregulation of miR-430 expression that could be partially explained with a reduction of its copy numbers in the genome.

## Introduction

Life-history is a vital force in the evolutionary shaping of embryonic development. Extreme examples are seasonal species where embryos undergo diapause, a suspension of development or dormancy state, as it is the case for the larvae of many insect species from a temperate climate. The seasonal life cycle has also evolved in a clade of teleost fishes (suborder Aplocheiloidei) known as annual killifishes. Annual killifish inhabit ephemeral bodies of water and are adapted to the alternation of wet- and dry season in Africa and South America. All adult fish die when their habitat dries out. The conservation of the species is ensured by desiccation-resistant eggs that enter in diapause and remain encased in the dry mud until the next rainy season. Annual life history is present in both South American and African killifishes and is the result of a series of independent events. Early studies suggested four events of loss and re-gain of an ancestral annual trait (Murphy and Collier, 1997; Hrbek and Larson, 1999), while more recent studies suggest that it repeatedly evolved, at least three times in Africa and three times in South America (Furness et al., 2015). In either of the two scenarios, an annual clade always has a sister non-annual clade that is phylogenetically closer than any other annual clade. The early development of annual killifish is conserved in the different lineages (Wourms, 1972; Furness et al., 2015; Dolfi et al., 2014) and is characterized by three points of developmental arrest. The primary point of developmental arrest occurs after the formation of the embryonic axis and organogenesis, during somitogenesis, and is called diapause II (DII). DII is a facultative stage: when embryos are incubated at high temperatures it can be skipped (Markofsky and Matias, 1977; Podrabsky et al., 2010; Furness et al., 2015; Valenzano et al., 2011; Blazek et al., 2013), but lower temperature, darkness or dehydration (all conditions occurring in natural habitats) induce DII (Podrabsky and Somero, 2007; Podrabsky et al., 2001; Levels and Denuce, 1988). The duration of DII is highly variable, and the embryos can remain in this stage for several months (Markofsky and Matias, 1977; Podrabsky and Hand, 1999) or even years (A.C., unpublished observation). The physiological and molecular mechanisms of diapause were studied in detail in the South American species, *Austrofundulus limnaeus*. Diapause is characterized by severe depression of protein synthesis, oxygen consumption, and mitochondrial respiration associated with G1 arrest of the cell cycle (Duerr and Podrabsky, 2010; Meller et al., 2012; Podrabsky and Hand, 1999; Podrabsky and Hand, 2000). These underlying mechanisms also seem to be conserved in the African annual genus Nothobranchius (Levels et al., 1986; Furness et al., 2015). Recent studies suggest that long-term organ preservation during diapause in killifish is regulated by the Polycomb complex protein CBX7 and other members of this protein family and a direct influence of vitamin D signaling in the induction of diapause (Hu et al., 2020; Romney et al., 2018).

Recent breakthrough studies have fundamentally transformed our understanding of diapause mechanisms. Singh et al. (2024) revealed that diapause evolved less than 18 million years ago in Killifish by co-opting ancient gene duplicates that originated more than 473 million years ago, identifying REST/NRSF and FOXO3 as critical transcription factors controlling the diapause gene expression program. This discovery establishes a new evolutionary framework showing that complex adaptations can evolve through regulatory remodeling of ancient genetic programs rather than through evolution of new genes.

The paradigm shift from viewing diapause as a passive state to understanding it as actively maintained through specific chromatin regulators has been established by Hu et al. (2020), who demonstrated that CBX7, a Polycomb complex member, is functionally required for muscle preservation and diapause maintenance. H3K27me3 chromatin marks are maintained at key developmental genes during diapause, providing the first mechanistic understanding of how complex organs are preserved during extended dormancy.

Studies in the roundworm *Caenorabditis elegans* have drawn a connection between diapause and aging. *C. elegans* can enter a stage of dormancy called dauer when the environmental conditions are unfavorable. Some genetic mutations that influence dauer formation also modulate longevity. In particular, the daf-2 mutation that affects an ortholog of the IGF/insulin receptor increases lifespan over two-fold. Strikingly, the influence of the IGF/insulin pathway on longevity is conserved in vertebrates and humans (Fontana et al., 2010; Kenyon, 2011). The gene expression profile in the dauer larvae stage shows high similarities to the expression profile of long-lived adult mutants (McElwee et al., 2004). In annual killifish, the expression of protein-coding RNAs during diapause and aging overlaps concerning genes controlling cell cycle and mRNA translation (Reichwald et al., 2015). Also, small non-coding RNAs are embedded in the genetic network that links diapause and longevity, as exemplified by miR-71. This miRNA is a longevity gene and an aging biomarker in *C. elegans* and is also essential for diapause (Zhang et al., 2011; Pincus et al., 2011; Boulias and Horvitz, 2012; De Lencastre et al., 2010). These results prompted us to investigate a possible overlap in miRNA regulation between aging and diapause in a vertebrate clade. We intensively study the short-lived fish *Nothobranchius furzeri*. This small annual killifish has a captive life span of a few months and is the vertebrate with the shortest life span that can be cultured in captivity. For these reasons, it is has emerged as a model organism of choice for biological investigations into aging (Harel et al., 2015; Ng’oma et al., 2014; Baumgart et al., 2014; Tozzini et al., 2013; Tozzini et al., 2012; Kirschner et al., 2012; Hartmann et al., 2011; Di Cicco et al., 2011; Valenzano et al., 2011). Next-generation sequencing (NGS) techniques can be used to identify and, at the same time, quantify miRNAs in non-model species taking advantage of the small size and the extremely high conservation of miRNAs sequences (Glazov et al., 2008; Bar et al., 2008; Morin et al., 2008). Using this technique, we described an evolutionarily conserved miRNA signature of aging in *N. furzeri* (Baumgart et al., 2012). More recently, based on the available genome sequence and an extensive database of small-RNA sequencing, we published a catalog of miRNAs in *N. furzeri* that contains both conserved and non-conserved miRNAs. An apparent possible function of the non-conserved miRNAs in this species may be to control diapause.

Aims of the present study were: i) to characterize the miRNA signature of DII investigating conserved and non-conserved miRNAs and ii) to compare this signature with miRNA regulation observed during aging. In order to characterize an evolutionarily-conserved signature of DII, we compared miRNA expression in embryos at mid-somitogenesis in pairs of annual and non-annual species from three independent evolutionary lineages of Aplocheloidei.

Our interspecies comparative approach is complementary to a recently published interspecies cross-sectional study where small RNA expression was analyzed in embryos of the South American annual killifish *Austrofundulus limnaeus* at different stages of development and in embryos that skipped diapause and proceeded to direct development.

## Results and discussion

### Phylogenetic sampling

**FIGURE 1:**
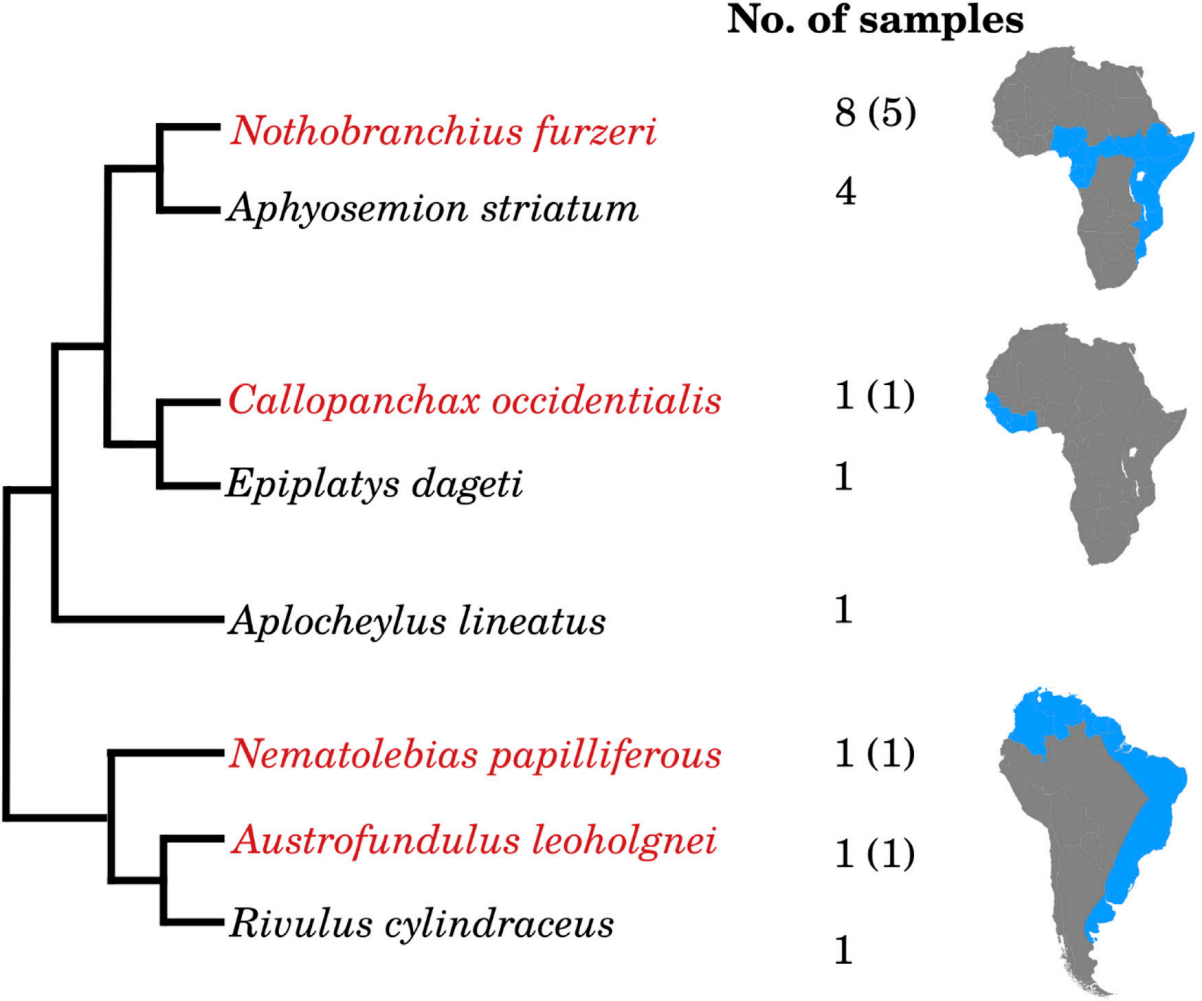
Overview of the sequenced killifish embryos and the corresponding number of samples. Fish marked in red are annual species, whereas all others are non-annual. For the annual species, the numbers of samples sequenced during diapause are given in brackets. *Aplocheylus lineatus* originates from India.

Annual killifish are divided into three main clades distributed in Africa (one West- and one East-of the Dahomey gap) and South America (Figure 1). In each of these regions, the clade contains both annual- and non-annual genera. The position of the non-annual genus Aplocheilus is debated. Earlier analyses considered it basal to all annual species (Murphy and Collier, 1997), but it was later suggested to be nested between African- and South American species (Furness et al., 2015). Therefore, Aplocheloidei offers a unique opportunity to study the parallel evolution of life-history adaptations (see also Dolfi et al., 2014). We collected eggs in diapause II from annual species or at the most similar morphological stage (24 somites) from non-annual species of all three clades, in addition to *Aplocheilus lineatus*, and analyzed miRNA expression by miRNA-Seq. We used as reference an updated version of the (*N. furzeri*) miRNA catalog we published previously (see, online Supplement Data 1). This catalog contains 754 loci containing 874 unique miRNA sequences, of which 498 belong to known conserved miRNA families and 376 to new, potentially killifish-specific miRNA families, *i.e.*, miRNAs that were only identified in *N. furzeri* so far. All killifish miRNA sequences were annotated against this reference, allowing two mismatches within the pre-miRNA sequence. From South America, we analyzed miRNA expression in the annual species *Austrofundulus leohoignei* and *Nematolebias papiliferous* and the related non-annual species *Rivulus cylindraceus*. From Africa, West of Dahomey gap, we analyzed the annual species *Callopanchax occidentalis* and the non-annual species *Epiplatys dageti monroviae*. From Africa, East of Dahomey gap, we analyzed the annual species *Nothobranchius furzeri* (5 replicates), and the non-annual species *Aphyosemion striatum* (4 replicates). Finally, we incubated eggs from *N. furzeri* (3 replicates) at a higher temperature, a condition known to promote direct development, *i.e.*, diapause skipping (Furness et al., 2015; Podrabsky et al., 2010).

### Overall expression activity of conserved and killifish-specific miRNAs

**FIGURE 2:**
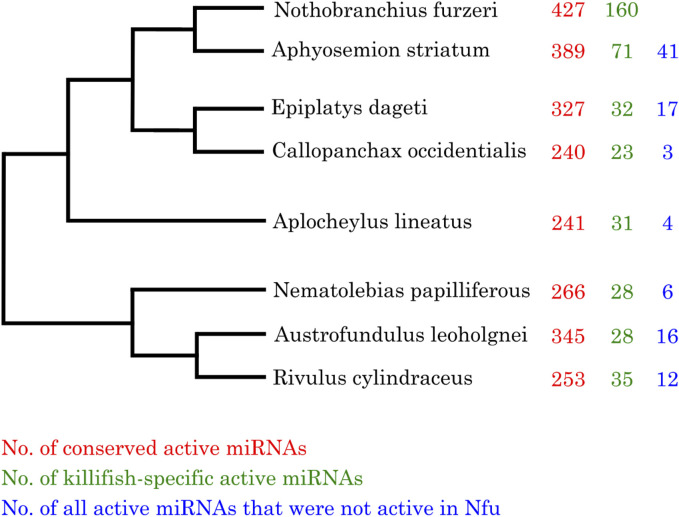
Actively transcribed conserved and killifish-specific miRNAs. Phylogenetic tree of the investigated killifish species and the number of their actively transcribed miRNA genes (red–miRNAs belonging a known miRNA family; green–miRNAs specific to killifish; blue–miRNAs not being transcribed in any of the *Nothobranchius furzeri* samples). Mature miRNAs having a count of more than ten reads were considered to be actively transcribed.

First, we investigated the total number of expressed miRNAs in all analyzed killifish species. As depicted in Figure 2, the largest number of expressed miRNAs, either conserved or killifish-specific, could be found in *Nothobranchius furzeri* and the more closely related species *Aphyosemion striatum*. For these species, multiple replicates were analyzed and higher sensitivity in *Nothobranchius furzeri* and the more closely related species was expected, because the killifish miRNA annotation was based on the *Nothobranchius furzeri* reference genome (Baumgart et al., 2017). Nevertheless, we observed the third-highest activity in miRNA genes in the more distantly related *Austrofundulus leohoignei* and the lowest number of active miRNAs in *Callopanchax occidentalis*, an African species. Most of these genes showed only low (10–50 reads) or moderate expression (51–1,000 reads). Combing all samples, we detected expression activity for roughly 45% of the predicted miRNAs genes described in our previous study (Baumgart et al., 2017). Interestingly, some predicted *Nothobranchius furzeri* miRNA genes did not show active transcription in any of the *Nothobranchius furzeri* samples. But were expressed in the other investigated killifish species, particularly in those not undergoing diapause. In particular, 41 miRNAs (19 conserved and 22 killifish-specific) were expressed in *A. striatum* corresponding to 8% of the total number of miRNAs expressed in this species. Out of the killifish specific miRNAs not expressed in *N. furzeri*, seven are expressed in *E. dageti*, one in *A. lineatus* and seven in *R. cylindraceus.* This result indicates that some miRNA genes present in the common ancestor of killifish are preserved, but transcriptionally inhibited in the investigated *N. furzeri* embryonic stages.

### Expression of miRNAs can separate killifish based on their diapause status

**FIGURE 3:**
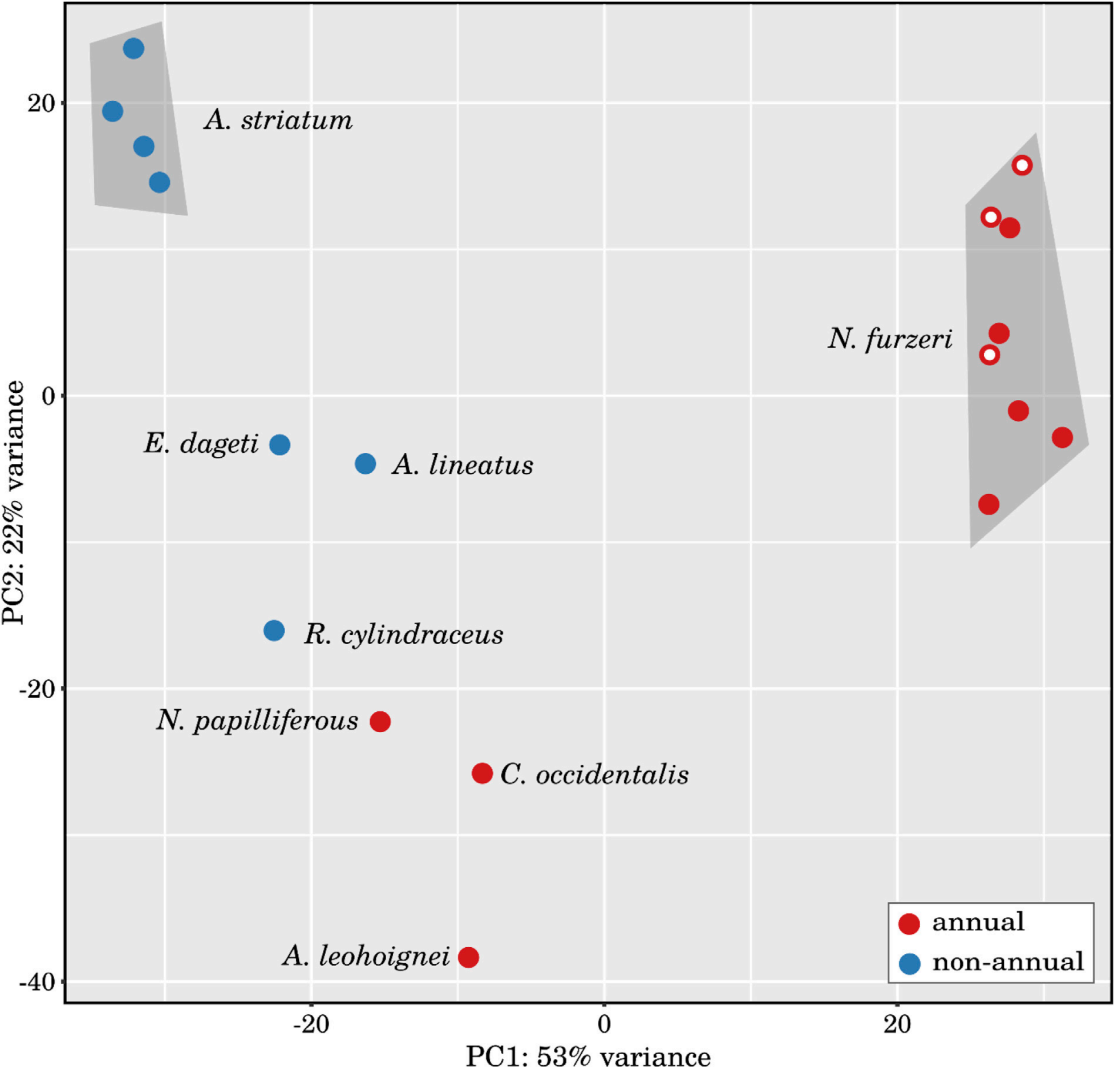
Principal component analysis of the investigated small RNA samples. PCA of the 18 RNA-Seq libraries based on the differentially expressed miRNAs identified in the *large interspecies comparison* (comparison I). The libraries generated from annual and non-annual species are relatively well separated and samples from *Nothobranchius furzeri* and *Aphyosemion striatum* cluster together, respectively. Samples from *N. furzeri* embryos that skipped diapause (marked with a white dot) show a visible shift in PC2 towards the *A. striatum* cluster.

Based on the global expression patterns of all miRNAs, we have clustered the analyzed samples using principal component analysis (see Figure 3) and heatmaps (see, online Supplement Data 4) and observed a clear separation not only between the individual species, but also between annual and non-annual embryos. Replicates of *Aphyosemion striatum* and *Nothobranchius furzeri* form defined clusters, whereas the three diapause-skipped *Nothobranchius furzeri* embryos are only weakly clustered. Nevertheless, these results demonstrate that samples are divided by their physiological status in addition to their phylogenetic relationships, indicating the existence of a convergent transcriptional program associated with diapause across species (see, online Supplement Data 4-6).

### Conserved expression of developmental-related miRNAs

**FIGURE 4:**
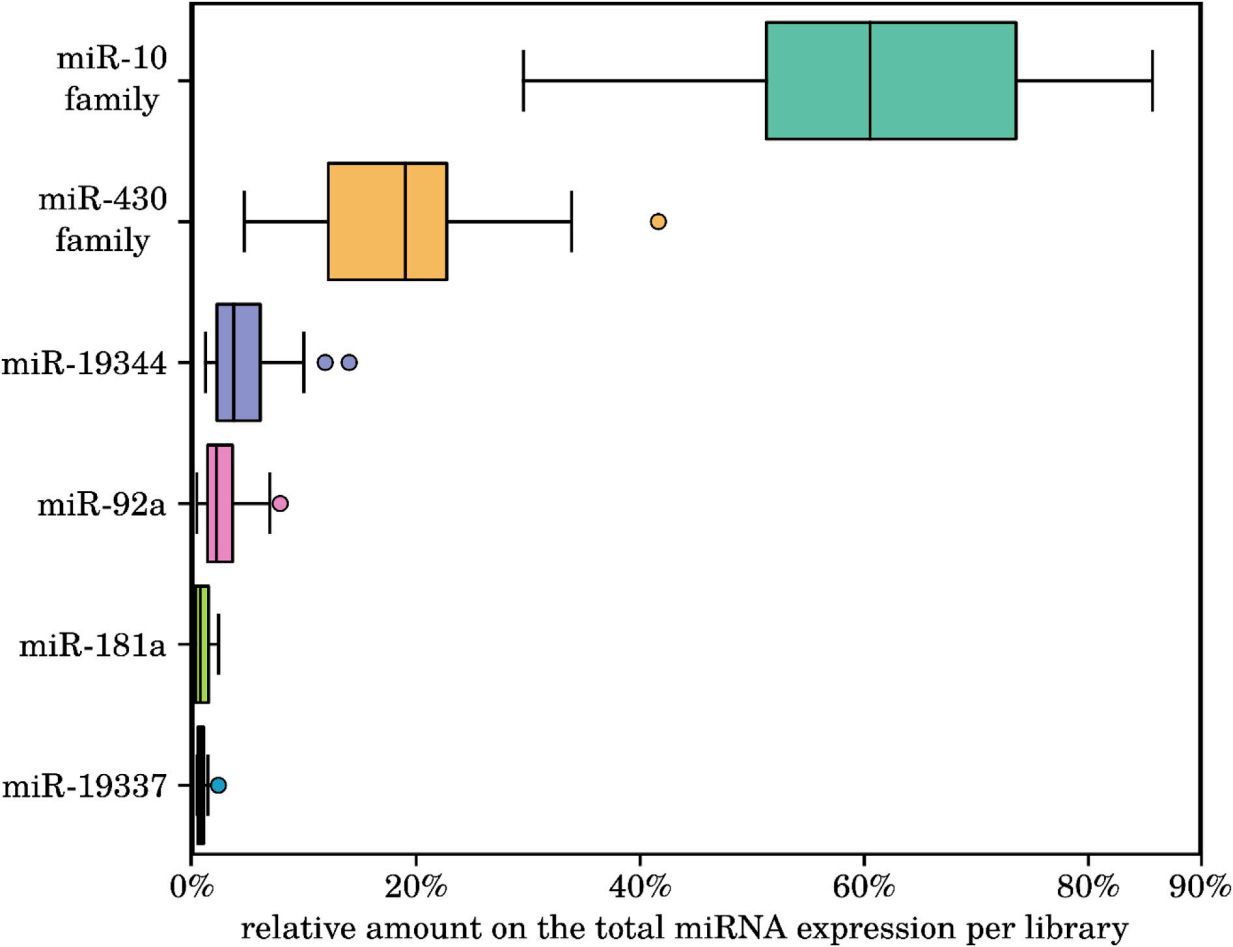
Relative amounts of the total expressed reads within all 18 sequenced killifish samples of the six most highly expressed miRNA families. The conserved miRNAs of the miR-10 and miR-430 families, miR-92a, miR-181a, and the two killifish-specific miR-19344 and miR-19337 showed the highest relative expression in all investigated samples. All of these miRNAs are known or predicted to play critical roles in the regulation of developmental processes in embryonic cells. For details on the miRNA expression levels, see online supplement data 1.

Next, we identified the most abundant miRNAs across all investigated samples. Interestingly, four conserved miRNA families (miR-10a/b/c, miR-92a, miR-181a and miR-430a/b/c) and two killifish-specific miRNA families (currently named miR-19337 and miR-19344) were always the most highly expressed in all the species. This observation may indicate a preeminent role during the early development of the investigated fishes (see, Figure 4 and online Supplement Data 1). This finding is consistent with the report of Romney and Podrabsky (2018), who also report these four conserved miRNA families to be the highest-expressed in embryos of the South American annual killifish *Austrofundulus limnaeus* at mid-somitogenesis. Of particular interest is the high expression of the two killifish-specific miRNAs miR-19337 and miR-19344, because this indicates that their evolution is linked to the control of some aspects of embryonic killifish development.

High expression of members of the miR-10 family is not unexpected as they are known regulators of several *hox* genes, which are essential transcription factors during embryonic development and particularly for the anterior-posterior patterning during somitogenesis (Woltering and Durston, 2008). Indeed, the expression of miR-10 sharply increases during somitogenesis in the annual killifish *Austrofundulus limnaeus* (Romney and Podrabsky, 2018). Additionally, it is also known that the expression of miR-10 has a positive effect on the ribosomal protein synthesis machinery (Ørom and Nielsen Finn Cilius, 2008), which is vital for an accurate progression of embryonic development (Amsterdam et al., 2004; Chakraborty et al., 2009; Palasin et al., 2019). Members of the teleost-specific miR-430 family are also key mediators of early embryogenesis. In the initial phase of maternal to zygotic transcription they cause degradation of maternal transcripts. In addition, they regulate other early developmental processes, such as meso-endodermal fate specification, brain morphogenesis and development of primordial germ cells (Rosa and Spagnoli Francesca, 2009; Tani et al., 2010; Mishima et al., 2006; Giraldez et al., 2005). Usually, miR-430 premiRNAs are produced by transcription of exceptionally large genomic clusters that contain multiple copies of the same miRNA and whose organization differs between teleost species (Baumgart et al., 2017). Recent work in *Austrofundulus limnaeus* has shown that expression of miR-430 is downregulated during somitogenesis and it is higher in embryos that escape diapause and thus proceed to direct development, underlining its broad relevance for the embryogenesis of teleost for both conserved and lineage-specific patters (Romney and Podrabsky, 2018).

Also, miR-181 is necessary for embryonic development, and the deletion of all its paralogs induces embryonic lethality (Pekarsky et al., 2006; Gibert et al., 2014). Besides, miR-181 targets another *hox* gene, namely, *hox-a11* in mammals, and the homeobox transcription factor Prox1, therefore being a regulator of embryonic development hinting at a potential similar function in fish embryos (Naguibneva et al., 2006; Kazenwadel et al., 2010).

The miR-92 is a member of the miR-17/92 cluster and an important well-studied positive regulator of the cell cycle (Uziel et al., 2009) and therefore cell proliferation during embryonic development.

For both killifish-specific miRNAs, target mRNAs are only predicted from sequence analysis in *Nothobranchius furzeri* (Baumgart et al., 2017). Among these, TSC2 and FAM83D are both putative targets of miR-19344. TSC2 is of particular interest as it is part of the TSC complex, a major upstream negative regulator of the activity of the mTORC1 complex that represents a central regulatory hub that integrates nutrient sensing and growth-factor signaling to regulate cell growth, protein synthesis, and cell proliferation (Orlova and Crino, 2010). mTORC1 is also a known major regulator of aging and longevity in multiple organisms (Thomas, 2018). FAM83D, the other potential target of miR-19344, is involved in cell proliferation and motility (Wang et al., 2013).

Relevant potential targets of miR-19337 are CECR2, being part of a protein complex that regulates neurulation, STARD13B a known inhibitor of cell growth (Ching et al., 2003) and SRF (Serum response factor), which modulates the expression of many immediate-early genes and therefore is an essential key player in embryonic development (Dalton et al., 1993).

From these examples, we can deduce that the developmental stage of the sampled embryos has a strong impact onto the expression of their respective miRNomes and highly abundant miRNA are also those that regulate developmental or associated processes. Especially the dominating abundance of miR-10 and miR-430 genes in almost all samples indicates the specialized roles of both miRNAs as well as their evolutionarily conserved regulatory importance for teleost embryogenesis.

### Differentially expressed miRNAs related to diapause regulation in killifish

To identify differentially expressed mature miRNAs (DEMs) linked to diapause regulation of annual species, we set up three different comparisons of the examined small RNA-Seq samples: (I) the *“large interspecies comparison”* contrasting all diapause samples from annual killifish against the non-annual killifish-derived samples, (II) the *“intraspecies comparison”* contrasting the *Nothobranchius furzeri* samples that underwent diapause against those that skipped it and (III) the *“selected interspecies comparison”* comparing the *Nothobranchius furzeri* diapause samples against the samples of its sister non-annual taxon, *Aphyosemion striatum*. Mature miRNA may arise from either the 5′- or 3′- side of the stem-loop that originate from a single miRNA gene and these forms target different mRNAs. Therefore, we have examined the mature 5′-p and 3′-p miRNAs of single miRNA genes separately in the subsequent analyses.

**FIGURE 5:**
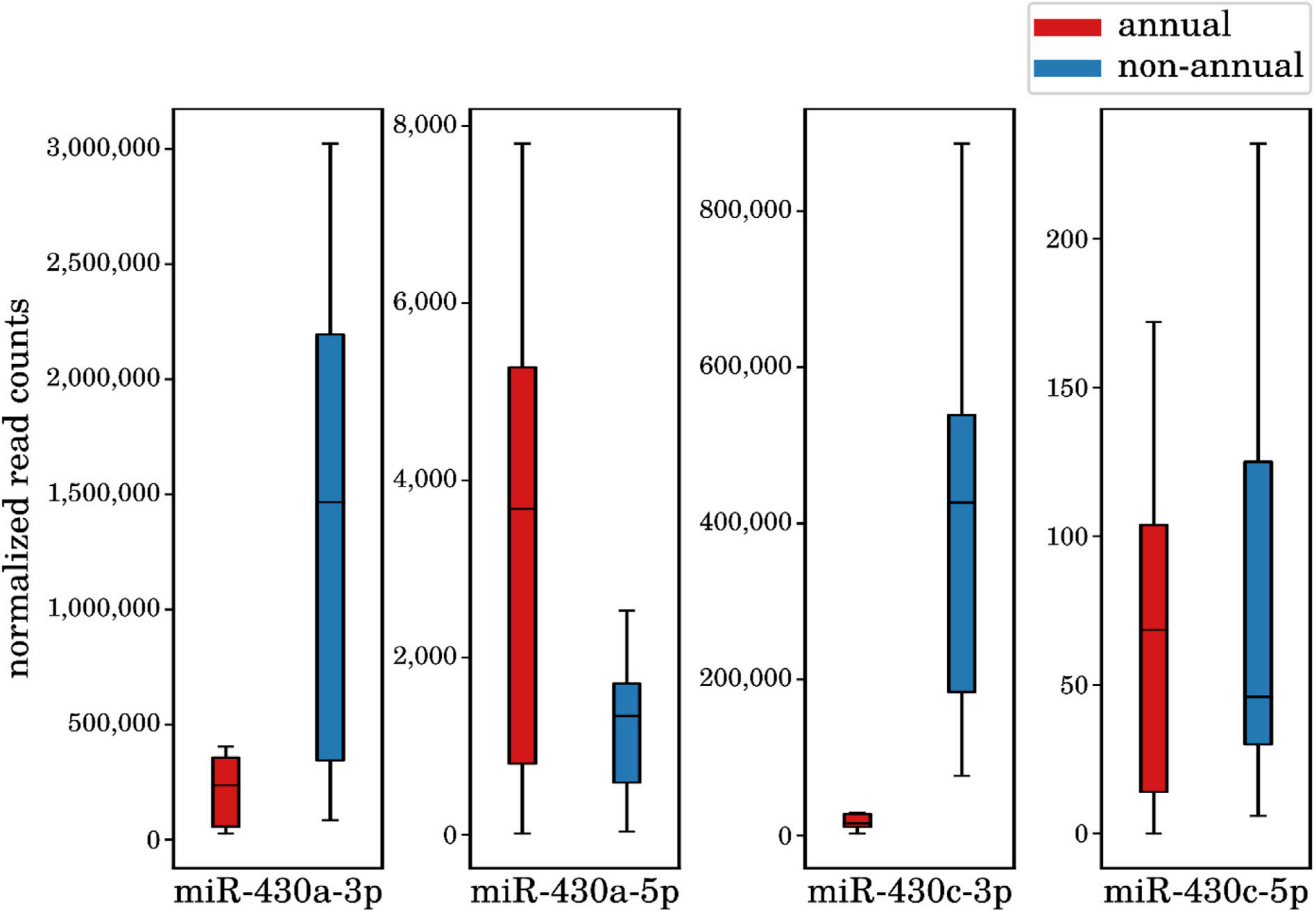
Expression differences of the miR-430a/c mature miRNAs between annual diapause (n = 8) and non-annual (n = 7) samples. The miR-430a/c-3p mature show a significant downregulation in all the annual species in diapause (p-Value 4.7*e*−8 and 4.2*e*−9, respectively), whereas its mature complementary form miR-430a/c-5p shows an upregulation in the same species (p-Value 7.9*e*−7 and 5.2*e*−2, respectively).

The *large interspecies comparison* (comparison I) detected 242 DEMs (122 upregulated and 120 downregulated in the annual killifishes compared with the non-annual ones) belonging to 156 distinct miRNA loci (see online supplement data 4). Some of the most significantly changing miRNAs in the annual comparison were members of the miR-430 family, essential regulators of developmental processes, and belonging to the above mentioned highest expressed miRNA genes. Interestingly, we found that the particular miR-430a/c-3p mature miRNAs are downregulated in all the annual species, whereas its mature complementary form miR-430a/c-5p is upregulated in the same species (see, Figure 5). This observation is striking, since the mature 5p transcripts are expressed at much lower levels as the corresponding 3p mature transcripts. This finding is a prominent example of a miRNA mature isoform switch that we describe in more detail in the next subsection. All the mature miRNAs of miR-430a and miR-430c act in developmental processes but target different mRNAs (Rosa and Spagnoli Francesca, 2009; Tani et al., 2010; Mishima et al., 2006; Giraldez et al., 2005). In addition to miR-430, we identified other DEMs with essential development-related functions, such as miR-19 being part of the miR-17/92 cluster and associated with endothelial cell differentiation in embryonic stem cells (Treguer et al., 2012) and more generally with the positive control of the cell cycle. Its downregulation is therefore expected given the profound depression of cell cycle in diapausing embryos (Dolfi et al., 2019). Similarly, miR-200 regulating the epithelial/mesenchymal transition during embryonic development (Gregory et al., 2008) and miR-221, which is involved in cell proliferation and angiogenesis (Urbich et al., 2008; Nicoli et al., 2012). All of these miRNAs seem to be necessary for the proper regulation of different developmental processes and may in part be responsible for the carefully orchestrated diapause regulation and embryogenesis in killifish.

The *intraspecies comparison* (comparison II) between the *Nothobranchius furzeri* samples in diapause and those that have skipped it due to high-temperature incubation, revealed 17 DEMs (10 up- and seven downregulated in the samples undergoing diapause) belonging to 14 distinct miRNA loci (see online supplement data 5). Observing only a few differentially regulated mature miRNAs between the diapause and non-diapause embryos of *Nothobranchius furzeri* can be due to the fact that not all embryos incubated at high temperature necessarily skip diapause and the samples are not pure samples on direct-developing embryos, but may also indicate a more simple miRNA control layer of diapause at least within this species. Prominent is the upregulation of miR-9-3p and miR-29a-5p during diapause. MiR-9 is an important regulator of neurogenesis (Coolen et al., 2013) by balancing quiescent and activated state of neuronal progenitors (Katz et al., 2016). The miR-29 family, on the other hand, is strongly upregulated during development and promotes neuronal differentiation (Liston et al., 2012; Napoli et al., 2020). In addition, both miRNAs are regulated during aging of *N. furzeri*, making them promising key regulators for diapause (Baumgart et al., 2012; Shioya et al., 2010). Most interestingly, the killifish-specific miR-19344 shows a 10-fold upregulation within the *intraspecies comparison* (comparison II). As discussed already above, one of its potential targets is TSC2, regulating the mTORC1 complex, which is an important modulator of cell growth, protein synthesis, and cell proliferation (Orlova and Crino, 2010). This finding strongly indicates that miR-19344 evolved in the killifish lineage as part of the molecular adaptations that enable diapause. Not much is known about the other DEMs of this comparison or their absolute change in expression is relatively low: One study suggests that miR-7641 is involved in endothelial differentiation in embryonic stem cells (Yoo et al., 2013) (this miRNA was differentially expressed in the annual comparison too), a recent study links miR-563 to the development of the spine *in vitro* (Zhang et al., 2017) and expression of miR-210 appears to promote angiogenesis by inhibiting *efna3*, a suppressor of blood vessel formation (Hu et al., 2010).

Within the *selected interspecies comparison* (comparison III), we observed a total of 292 DEMs (171 upregulated and 121 downregulated in the annual *Nothobranchius furzeri* samples compared with the non-annual *Aphyosemion striatum*) belonging to 203 distinct miRNA loci (see online supplement data 6). We identified a large proportion of DEMs in common between comparison I and comparison III, including the same directional changes in expression (see online supplement data 7). This overlap includes the miR-430, miR-7641-3p, miR-10, and miR-17 mature miRNAs discussed above. Still, we identified unique DEMs belonging to 25 and 68 miRNA loci in the *large interspecies comparison* and the *selected interspecies comparison*, respectively. Interestingly, we found both mature miR-19344 forms upregulated within the annual samples compared to the non-annual samples. Those miRNAs unique to the *Nothobranchius furzeri* and *Aphyosemion striatum* comparison might be in part responsible for the difference in embryogenesis in their clade but not necessarily in the other annual/non-annual killifish clades, because annual life appears to have evolved independently several times (Murphy and Collier, 1997; Hrbek and Larson, 1999; Furness et al., 2015).

In general, the regulatory involvement of miRNAs during the embryogenesis of killifish seems to be pervasive. In contrast, only a few miRNAs seem to be involved in the maintenance of diapause in *Nothobranchius furzeri*, making these miRNAs critical regulators of this dormancy state.

### Mature miRNA switch

**FIGURE 6:**
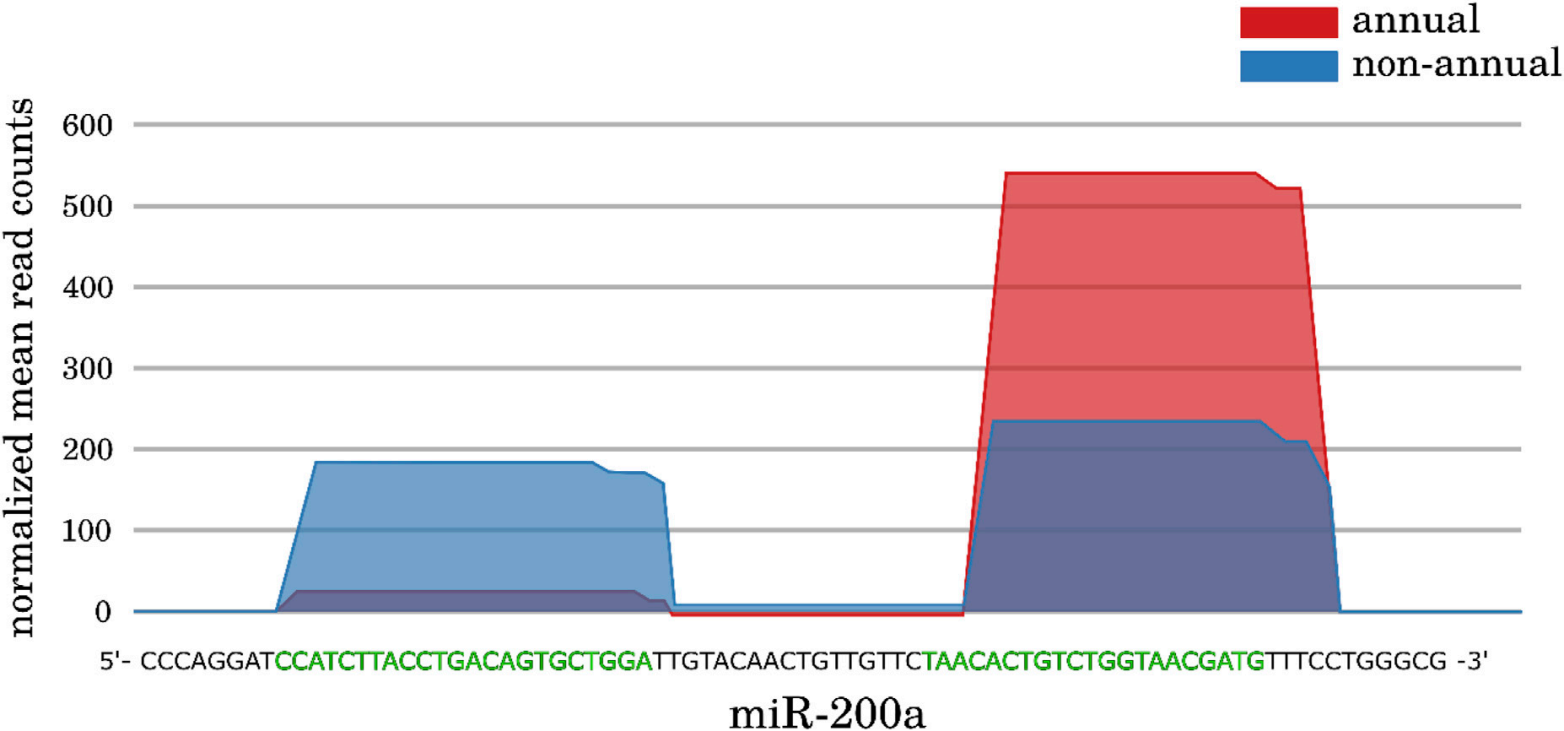
Expression profiles of the miR-200a gene from the *large interspecies comparison* (comparison I). No significant change in expression can be observed when comparing all reads of the pre-miR-200a annotation between both conditions (p-Value = 0.43). However, a clear difference can be observed for the 5p and 3p mature miRNAs separately (p-Value of 1.0*e*−9 and 2.1*e*−4). In contrast to the non-annual *A. striatum* (n = 4) were both mature sites are equally expressed, the 3p mature miRNA is increased in expression, whereas the 5p mature miRNA appears to be “switched off” within the annual *N. furzeri* (n = 5). The green bases indicate the mature sequences. For more examples, see online supplement data 3.

Within our differential expression analysis, we observed some interesting cases of DEMs. In particular, we found some miRNA genes that showed a “switch” in the expression of their main mature miRNA: We did not observe a change in the total transcribed amount of the pre-miRNA transcripts between contrasted conditions, but in the relative abundance of the 5p and 3p mature miRNAs. The reason could be an alteration of the processing machinery responsible for the biogenesis of the mature miRNA products. In addition to the miR-430 family, we identified two such cases in comparison I (miR-200a and miR-181a) and four in comparison III (miR-128, miR-130c, miR-217 and miR-223), for an example see Figure 6 and online supplement data 3. We already briefly mentioned the regulatory targets of miR-200a and its potential role during diapause in the previous subsection. But also the mature miRNA switch cases show potential regulatory roles in different maturation processes: Developmental regulation of T cells and the vascular system is modulated by miR181a (Li et al., 2007; Kazenwadel et al., 2010), miR-217 shows strong expression in late stages of the fetal rat development and an expression disruption showed damaged lung tissue (Bhaskaran et al., 2009), and miR-223 is a well-studied modulator in hematopoiesis and osteoclast differentiation (Felli et al., 2009; Fazi et al., 2005; Sugatani and Hruska, 2007). The functions of miR-128 are more linked to the formation and progression of various cancers, suggesting a general role in either cell development or proliferation, and it is known to inhibit telomerase activity (Huang et al., 2018; Lin and Wu, 2018; Guzman et al., 2018). Not much is known about miR-130c; however, its paralog miR-130a appears to be present in hematopoietic progenitor cell but not in mature blood cells (Escobar et al., 2014).

### Strong correlation between the identified differentially expressed miRNAs and expression levels of developmental-related mRNAs

Recent transcriptomic analysis by Clouser et al. (2025) has revealed that diapausing embryos of Austrofundulus limnaeus mount a robust transcriptomic response within 24 h of anoxic exposure, with over 500 differentially expressed transcripts. This study demonstrates that diapause and anoxia-induced quiescence represent distinct physiological states, contradicting previous assumptions that diapausing embryos are metabolically inert. The research reveals upregulation of genes involved in integrated stress response, lipid metabolism, p38mapk kinase signaling, and apoptosis during anoxic exposure.

Here, we examined to which extent the results from the differential expression analysis of the miRNAs are reflected on the transcript regulation level. To do so, already identified aging-related differentially expressed genes (Reichwald et al., 2015) were compared with the predicted targets of the identified DEMs. We found that 143 DEMs have 88 unique mRNA targets that show a significant expression change within the *selected interspecies comparison* (see online supplement data 6). If we look at targets of some of the DEMs discussed above, we see a clear overlap to the identified differentially expressed mRNAs. Almost all of them are associated with cell developmental or proliferation processes and show opposite expression patterns compared with their targeting miRNAs. This observation indicates a strong correlation between the expression of the identified DEMs and the regulation of their target mRNAs. As one example, cyclin D1 is one of the targets of the DEM miR-430a/c-3p, promoting G1 progression of the cell cycle (Baldin et al., 1993) and was found differentially expressed.

Additionally, for each DEM, we tested if there was a specific enrichment of its targets among the DEGs and could identify 23 miRNAs where this was the case. Some of the most significant enrichments were observed for miR-23a-3p, miR-731-5p, miR-17a/b-3p, miR-430a-5p and miR-19b-5p, all of them discussed already above (see online supplement data 8).

Pathway analysis of the predicted targets of DEMs for the *interspecies comparisons* shows suppression of cell cycle/DNA replication and nervous system development during diapause (see online supplement data 4 and 6).

### The expression of miRNAs involved in embryogenesis and diapause regulation is modulated in an aging related manner

The identification of miR-29 as a critical driver of aging-related phenotypes across vertebrate species provides important context for understanding diapause-aging connections. Studies demonstrate that miR-29 overexpression is sufficient to drive aging-related phenotypes and leads to early lethality, while partial loss extends lifespan in progeria models. This finding significantly advances our understanding of microRNA control of aging processes that should be integrated with diapause research.

Comparative analysis across mammalian embryonic diapause by Iyer et al. (2024) demonstrates that microRNA activity is essential for the molecular rewiring required to establish dormancy, with individual miRNAs contributing to combinatorial regulation networks. This research identifies TFE3 as an upstream regulator of diapause-specific miRNAs, linking cytoplasmic mTOR activity to nuclear miRNA biogenesis, providing important comparative context for killifish studies.

Our previous work revealed a correlation between transcriptome regulation during diapause and aging (Reichwald et al., 2015). Here, we extend this analysis to miRNA regulation. We found miR-29a-5p and miR-29a-3p to be similarly upregulated during diapause and aging within the aged brain, liver, and skin samples of *Nothobranchius furzeri*. The miR-29a gene was already known to be upregulated in highly aged individuals and is also considered a tumor suppressor (Baumgart et al., 2012). Another upregulated miRNA in both conditions was miR-210, which acts in the hypoxia pathway (Xin and Le Quynh-Thu, 2010). When active, this miRNA positively regulates the adaptation to a hypoxic environment, helping cells to survive with only low available oxygen (Xin and Le Quynh-Thu, 2010). Diapausing killifish embryos show severe repression of aerobic metabolism (Podrabsky and Hand, 1999; Duerr and Podrabsky, 2010) and are incredibly resistant to hypoxia (Podrabsky and Somero, 2007); therefore, under normal oxidative conditions, hypoxia-sensitive genes may be downregulated. Hypoxia is also a known condition in aged tissues, indicating that miR-210 is generally used to respond to this oxidative stress (Jebessa et al., 2018). We observed the expression of miR-222a to be equally and strongly downregulated during diapause and aging. When active, miR-222a promotes the expression of proteins responsible for muscle cell development by silencing the expression of the translational repressor *cpeb3* (Zhang et al., 2011). However, neither miR-9 nor the killifish-specific miR-19344 could be correlated with aging and seem to be specific for diapause and early developmental regulation. When including DEMs of the *selected interspecies comparison* (comparison III), we observed a considerable overlap of 57 and 40 DEMs being up- and downregulated in the same way during embryogenesis and aging. Additionally, we found 99 DEMs being modulated in opposite directions (see online supplement data 7). Closer inspection revealed unusual behavior of some of the already discussed miRNAs. Both 3p mature transcripts of miR-430a/c are downregulated during diapause but upregulated during aging in the brain. In the aged brain and liver of *Nothobranchius furzeri*, miR-10b-5p is downregulated, whereas it is upregulated in embryonic development. The same is true for miR-92b-3p in the liver and skin. Also interesting, miR-181b-3p is downregulated in both diapause and aging, but miR-181b-5p appears to be upregulated during diapause but stays downregulated in the aged liver and skin samples and not significantly changed in the brain during aging.

These exemplarily described behaviors of specific miRNAs already indicate that there is an overlap between aging processes and the regulatory network controlling diapause and embryogenesis in *Nothobranchius furzeri*. However, to elucidate the precise mechanisms or regulatory responses that act in both conditions, more functional analyses need to be performed.

### Evolution of diapause is linked to a contraction of miR-430 family copy numbers

**FIGURE 7:**
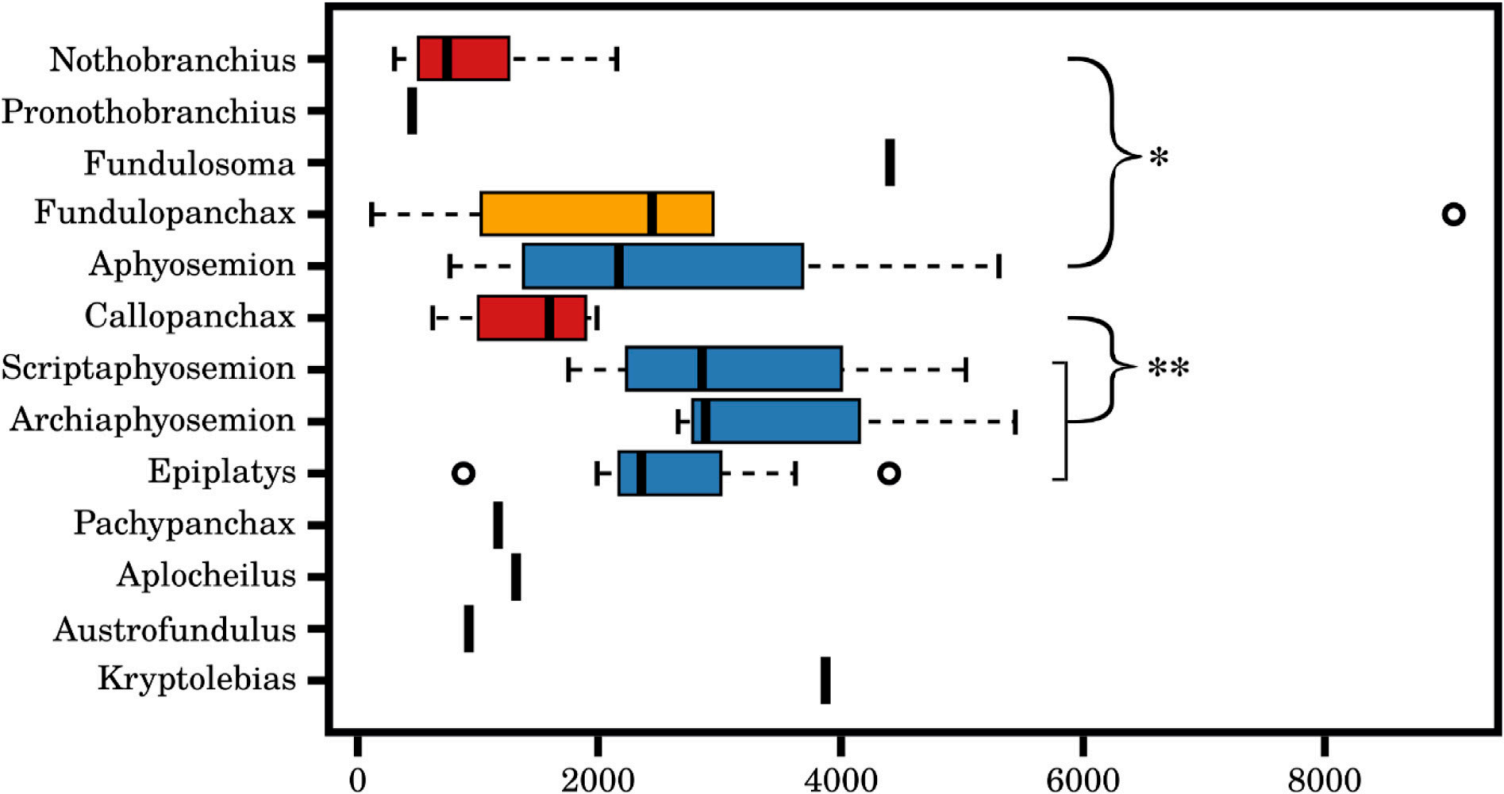
Difference of genomic miR-430 copy number within different killifish species. Estimated number of miR-430 gene copies within different killifish species based on Illumina short read data. A significant difference between the annual genera *Nothobranchius* (n = 10) and the non-annual genera *Aphyosemion* (n = 9) (Wilcoxon rank sum test: W = 11, p-value = 0.002089), as well as, between the annual genera *Callopanchax* (n = 4) and the non-annual genera of *Scriptaphyosemion* (n = 4), *Archiaphyosemion* (n = 2), and *Epiplatys* (n = 7) (Wilcoxon rank sum test: W = 5, p-value = 0.01176) were observed. The miR-430 gene copy number is lower in annual genera (red) than semi-annual genera (yellow) or non-annual genera (blue).

The miR-430 locus in teleost fishes is large, variable across species, and characterized by many repetitions of its family members. We reasoned that differential expression of miR-430 family members in the embryos of annual and non-annual killifish could be associated with variations in the copy number of miR-430 stem-loops. To test this hypothesis, we took advantage of the recently sequenced genomes of *Nothobranchius orthonotus*, *Aphysosemion australe*, *Callopanchax*, and *Pachypanchax platyfairii* and the low-coverage genomes of 45 annual and non-annual killifish species from Africa. Highly repetitive loci are difficult to assemble from short-reads and thus may be under-represented in genome assemblies. To obtain an unbiased estimate of copy numbers, we, therefore, quantified the fraction of reads that match to any sequence of the miR-430 family (two mismatches allowed) in these different genomes, accounting for genome size. Although this approach only provides a crude estimate of copy number in each species, its precision should allow a comparison between different genera, which contain multiple species. The apparent result is that the copy number is lower in the annual genera *Nothobranchius* (N = 10) and *Callopanchax* (N = 4) as opposed to the non-annual genera *Aphyosemion* (N = 9), *Scriptaphyosemion* (N = 4), *Archiaphyosemion* (N = 2), and *Epiplatys* (N = 7) (see Figure 7). Only two genomes are available for the African clade, the annual *Austrofundulus limnaeus* and the closely related non-annual *Kryptolebias marmoratus*. Similarly, in this case, the difference is in the expected direction. The semi-annual genus *Fundulopanchax* showed an estimated copy number distribution intermediate between the non-annual genus *Aphyosemion* and the annual genus *Nothobranchius*, although they do not differ statistically.

## Conclusion

In the present paper, we provide one of the first miRNA comparative studies of vertebrate diapause. We could observe that regulation of miRNA expression in different clades of annual killifishes shows convergent evolution, and the samples of different taxa do not cluster according to their phylogenetic relationships, but according to their physiological status (*i.e.*, being in diapause or not). This observation represents a further example of how annual killifish, belonging to different clades, independently converged on similar adaptations to support embryonic development in their ephemeral habitat (Dolfi et al., 2014; Furness et al., 2015). Additionally, we describe different miRNAs as key modulators during fish embryogenesis and diapause regulation, such as members of the miR-10 and miR-430 family, miR-92, and miR-29a, but also killifish-specific ones, such as miR-19344 and miR-19337. Some of these miRNAs are of particular interest because their implications in specific developmental processes are already further studied in other species. For example, miRNA-430 acts primarily by inhibiting protein synthesis (Bazzini et al., 2012). However, after the onset of zygotic transition and in adult cells, mRNA degradation appears to be the predominant mechanism (Eichhorn et al., 2014). Diapause is characterized by a prominent depression of protein synthesis that is reduced to 10% of the pre-diapause levels (Podrabsky and Hand, 2000). It is therefore highly likely that both proteins and mRNAs are stabilized during diapause. In this context, miRNAs should act primarily, if not exclusively, by inhibiting protein synthesis. A prominent feature of miRNA regulation during diapause II is a dramatic downregulation of miR-430 expression. This is paralleled by a reduction of the miR-430 copy numbers in the genomes of annual killifish, and this reduction may well represent the basis for this downregulation. Upon exit of diapause, the release of miRNAs would allow the immediate onset of translation. This concept is further supported by the observation that diapausing embryos of *Nothobranchius furzeri* show paradoxical upregulation of genes related to translational elongation (Reichwald et al., 2015), suggesting that these embryos are primed for a catch-up process upon exit from diapause.

Studies in the worm *Caenorhabditis elegans* revealed miRNAs that are regulated both during diapause and aging. In particular, miR-71 does not influence diapause entry but is necessary for survival during diapause in *Caenorhabditis elegans* (Zhang et al., 2011). Deletion of miR-71 results in a shortened lifespan, and miR-71 abundance increases with age before dropping late in life (De Lencastre et al., 2010). The timing of the miR-71 expression drop can be used to predict the longevity of individual worms (Pincus et al., 2011). Our results show a similar overlap between miRNAs regulated during diapause and aging in the annual killifish *Nothobranchius furzeri*. Several miRNAs significantly modulated in diapausing *Nothobranchius furzeri* were modulated during aging as well. One of these miRNAs, miR-101a, is particularly interesting. He *et al.* showed that miR-101 acts as a highly connected hub in gene regulatory networks of transcription factors and epigenetic modulators involved in cell cycle progression (He et al., 2012). Overexpression of miR-101 is also known to induce cell cycle arrest in different cell types (He et al., 2012). So, high levels of miR-101 in diapausing embryos would contribute to the G1 block typical of diapause (Meller et al., 2012).

On the other hand, we observed downregulation of members of the miR-17/92 cluster, also known as oncomiR-1. The organization of this cluster is conserved in *Nothobranchius furzeri*. Furthermore, it was observed to be downregulated during the aging of the brain (Baumgart et al., 2012) and adult neuronal progenitors (Terzibasi Tozzini et al., 2014). OncomiR-1 is a significant regulator of cell cycle and overexpressed in several tumors (Olive et al., 2010). On the other hand, it is well established that oncomiR-1 is downregulated during the aging of human mitotically-active cells where it targets the cell cycle inhibitor p21 (Hackl et al., 2010). An overlap in the regulation of cell cycle protein-coding genes between diapause and aging was also demonstrated by RNA-seq analysis in *Nothobranchius furzeri* (Reichwald et al., 2015), further supporting the concept that, like in *Caenorhabditis elegans*, aging and diapause are controlled by overlapping genetic pathways. Our study results serve as a sound basis for further research to infer the impact and precise control of miRNA regulation of these overlapping pathways.

Current best practices for killifish miRNA analysis include ribosomal RNA-depleted RNA-seq for comprehensive transcriptomic analysis and dedicated small RNA-seq for microRNA profiling. Single-cell approaches are emerging as powerful tools for understanding cell-type-specific regulation during diapause transitions.

Recent advances necessitate new research directions including single-cell resolution studies of microRNA regulation during diapause transitions, functional validation using advanced CRISPR methods, and integration of chromatin and microRNA regulation through multi-omics approaches. The convergence of mechanistic studies in model organisms with human aging research promises to accelerate the development of interventions that promote healthy aging and combat age-related diseases.

## Materials and methods

### Fish raising

Fish were raised, fed, and bred as described in Dolfi et al. (2014). The protocols of fish maintenance were carried out in accordance with all animal use practices approved by the Italian Ministry of Health (Number 96/2003a) and the local animal welfare committee of the University of Pisa. All experimental procedures were managed, following the prescription of the European (Directive 2010/63/UE) and Italian law (DL 26/04-03-2014).

### RNA isolation

For each species, 20 eggs were pooled for each preparation. Total RNA was isolated using the miRNeasy Mini Kit QIAGEN according to the manufacturer’s protocol.

### Small RNA sequencing

For small RNA sequencing, small RNA cDNA libraries were prepared as follows: for each developmental stage, equal quantities (1 μg) of total RNA were submitted independently to Illuminas TruSeq small RNA sample preparation protocol (Illumina Inc., San Diego, United States). The library preparation was performed as follows: RNA was ligated with proprietary adapters to the 5′ and 3′ termini of the RNA. The adapter-ligated samples were used as templates for cDNA synthesis. The cDNA was amplified with 13 PCR cycles to produce sequencing libraries, introducing specific nucleotide codes for indexing libraries to allow multiplexed sequencing. The cDNAs were purified by 10% Novex TBE polyacrylamide gel electrophoresis (Invitrogen) and eluted into 300 μL elution buffer (Illumina) for at least 2 h at room temperature, to enrich for molecules containing inserts in the range of 18–33 nt. The resulting gel slurry was passed through a Spin-X filter (IST Engeneering Inc. Milpitas, CA, United States) and precipitated by the addition of 20 μg glycogen, 30 μL of 3M NaOAc, pH5.2, and 975 μL of pre-chilled (−20°C) ethanol. After washing with 70% ethanol, the pellet was dried at 37°C for 5–10 min and dissolved in 10 μL resuspension buffer (Illumina). The purified libraries were quantified on the Agilent DNA 1000 chip, diluted to 10 nM, and subjected to sequencing-by-synthesis on Illumina HiSeq 2000.

### RNA-seq analysis

Clipping the RA3 adapter of the TruSeq small RNA preparation kit (5′-TGG​AAT​TCT​CGG​GTG​CCA​AGG) and trimming the read ends by a quality threshold of 20 was performed with PRINSEQ (v0.20.3) (Schmieder and Edwards, 2011). Read quality was monitored by FastQC (v0.11.3; http://www.bioinformatics.babraham.ac.uk/projects/fastqc). Mapping was done using Hisat2 (v2.0.4) (Kim et al., 2015) onto the *N. furzeri* reference genome (Reichwald et al., 2015), for details see online supplement data 1. Read counting was done on the improved version of our published miRNA annotation of *N. furzeri* (Baumgart et al., 2017), counting reads for each mature miRNA of each pre-miRNA annotation separately. Analysis of differentially expressed miRNAs was performed by the Bioconductor package DESeq2 (v1.10.0) (Love et al., 2014) within three defined sample comparisons (see online supplement data 2). Only mature miRNAs having a count of more than ten normalized reads were considered to be actively transcribed and used for subsequent analyzes. The resulting p-values were adjusted using the Benjamini and Hochberg’s FDR approach (Benjamin and Hochberg, 1995). We considered mature miRNAs with an adjusted p-value of *<* 0.05 as differentially expressed. We did not use a fixed fold-change threshold because modest differences in expression can still be statistically significant (and *vice versa*). In our dataset, the smallest log_2_ fold change among differentially expressed miRNAs was 0.944 (approximately a twofold change), with most miRNAs showing log_2_ fold changes well above 1.0.

### Enrichment analysis of miRNA targets

We used the hypergeometric test to calculate enrichment scores of miRNA targets within the published set of differentially expressed aging-related *Nothobranchius furzeri* genes (Reichwald et al., 2015):

$$p-\text{value}=\frac{R!n!\left(N-R\right)!\left(N-n\right)!}{N!}\;\sum_{i=r}^{\min\left(n,R\right)}\frac{1}{i!\left(R-i\right)!\left(n-i\right)!\left(N-R-n+i\right)i},$$

where *N* is the total amount of known protein-coding genes in *Nothobranchius furzeri*, *R* the number of differentially expressed genes of the given set, *n* the number of protein-coding genes with predicted miRNA target sites, and *r* the number of differentially expressed genes with predicted miRNA target sites. Enrichment of individual miRNAs, in the set of aging-related genes, was calculated similarly, with *N* being the total number of protein-coding genes with predicted miRNA target sites and *n* the number of genes, containing a target site of the respective miRNA. The resulting p-values were adjusted using Benjamini and Hochberg’s FDR approach (Benjamin and Hochberg, 1995) and were considered significant if *p* was less than 0.05.

### Estimating miR-430 copy number from short genomic reads

Using raw Illumina short sequencing reads of 50 killifish species from previous studies (Cui et al., 2019; Wagner et al., 2018; Kelley et al., 2016), we counted the number of reads containing the following conserved motifs of eight isoforms of miR-430 identified in *N. furzeri* (UAA​GUG​CUA​UUU​GUU​GGG​GAA​G, UAA​GUG​CUA​ACU​GUU​GGG​GUA​G, UAA​GUG​CUA​ACU​GUU​GGG​GUA​U, UAA​GUG​CUA​CAU​GUU​GGG​GCA​G, CAC​CUC​AAA​GAA​UCC​ACU​GA, ACU​CUG​ACA​AAG​GCA​CUG​ACU, UAA​GUG​CUA​CAU​GUU​GGA​GUC​A, UAA​GUG​CUU​CUC​UUU​GGG​GUU​G), allowing for a maximum of two mismatches to account for possible divergence between species using a custom C++ program (https://github.com/melop/mirseek/), which depends on the edlib library. Genome sizes for these species were taken from previous studies. The estimated miR-430 counts per haploid genome can be computed as: ℎ(*lg*⁄), where *h* is the number of sequence hits, *l* the total read length, and *g* the genome size.

## Data Availability

All RNA-Seq samples analyzed in this study are deposited at the NCBI Geo or Bioproject database under the following IDs: GSE81231, GSE92854, PRJNA531796, PRJNA290522. All supplement data are available as online supplement data deposited at the Open Science Framework (https://osf.io/eqf79/).
